# Calcifying fibrous tumor of stomach: A case report

**DOI:** 10.1097/MD.0000000000008882

**Published:** 2017-11-27

**Authors:** Bo-jing Li, Xiao-dan Yang, Wei-xiang Chen, Yi-hai Shi, Zhi-hong Nie, Jian Wu

**Affiliations:** a Department of Gastroenterology; b Department of Radiology; c Department of Pathology, Gongli Hospital, Second Military Medical University, Shanghai, China.

**Keywords:** calcifying fibrous tumor, endoscopic submucosal dissection, gastrointestinal stromal tumor

## Abstract

**Rationale:**

Calcifying fibrous tumor (CFT) is a rare benign soft tissue mesenchymal neoplasm. Although the gastrointestinal (GI) tract is the most common predilection site of CFT, the clinicians, even including pathologist, generally consider it as GI stromal tumor (GIST) or other submucosal tumors such as schwannoma and leiomyoma.

**Patient concerns:**

A 55-year-old man presented with complaints of epigastric discomfort and abdominal distention for more than 1 year.

**Diagnoses:**

On the basis of endoscopic and computed tomography examination, preliminary diagnosis was GIST.

**Interventions:**

Endoscopic submucosal dissection (ESD) surgery was performed to remove the gastric mass.

**Outcomes:**

The histopathological examination revealed a gastric CFT.

**Lessons:**

We present a case of gastric CFT, which was misdiagnosed as GIST based on endoscopic and radiologic findings.

## 1 Introduction

Calcifying fibrous tumor (CFT) is a rare tumor that typically occurs in the limbs, trunk, neck, and deep soft tissue.^[1]^ With the development of medical imaging technology and improvement of clinicians’ understanding of this tumor, the majority of recently reported CFT cases occurred mainly in the gastrointestinal (GI) tract.^[2,3]^ The most frequently identified sites of GI tract were stomach, small bowel, and colon.^[3,4]^ CFTs have a female predominance and the mean patient age is 33.6 to 49.2 years.^[4]^ The occurrence of CFTs in the stomach was more common in older adults. Most CFTs are incidentally identified during endoscopic examination or unrelated surgical procedures. The best treatment for CFTs is local resection. Open surgical resection is performed in most cases of CFT, while laparoscopic or minimally invasive surgery was also used in some cases.^[5]^ Although endoscopic submucosal dissection (ESD) surgery is more suitable for CFTs, very few cases were reported to be excised with this method.^[6]^

## 2 Case presentation

**Figure 1:**
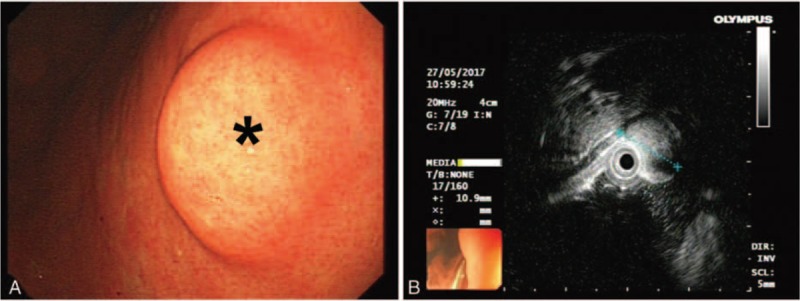
Gastroscopy indicated a semi-circular submucosal neoplasm of 2 cm on the posterior wall (as shown by the asterisk) of the stomach (A). Endoscopic ultrasonography showed a low-density lesion with distinct boundary, which was derived from the muscularis propria of the gastric wall (B).

**Figure 2:**
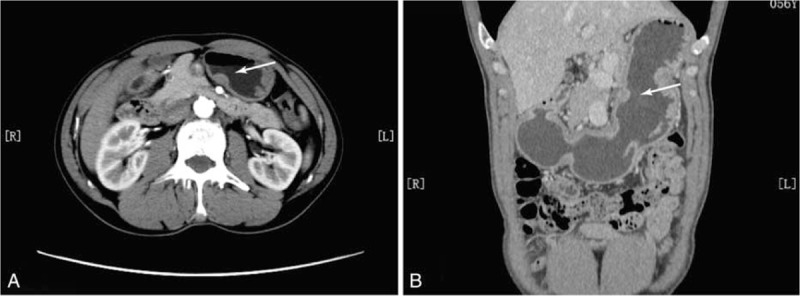
The abdominal enhanced CT scan indicated a nodule with similar density as soft tissue on the lesser curvature of the stomach (A). The CT coronal reconstruction displayed a submucosal tumor on the small curved side of the middle stomach region (B).

A 55-year-old man presented with complaints of epigastric discomfort and abdominal distention for more than 1 year. The patient's symptoms could voluntarily alleviate, and were unrelated to food intake or variety. The patient suffered from hypertension for 1 year and needed drugs to control the condition. Gastroscopy indicated a semi-circular submucosal neoplasm, which was on the posterior wall of the lower gastric region. It was about 2 cm in size with a smooth surface (Fig. 1A). Endoscopic ultrasonography showed a low-density lesion, 1.6 × 1.5 cm in size with distinct boundary, which was derived from the muscularis propria of the gastric wall (Fig. 1B). The abdominal computed tomography (CT) scan indicated a nodule with similar density as soft tissue on the lesser curvature of the stomach, and the CT value was 50 Hu. The abdominal enhanced CT scan indicated the CT value increased from 61 Hu of arterial phase (Fig. 2A) to 84 Hu of equilibrium period, which revealed a delay in the lesion's enhancement. The CT coronal reconstruction displayed a submucosal tumor on the small curved side of middle stomach (Fig. 2B). The clinicians considered the submucosal tumor as GI stromal tumor (GIST) of the stomach, which was the most common submucosal tumor of the digestive system. ESD surgery was performed to remove the mass. A small submucosal mass with a diameter of 1.8 cm was observed in the lesser curvature of the stomach. The combination of saline, epinephrine, and methylene blue was injected into the submucosa to expand it. After local incision of the mucosa with a Hook knife, a white tumor was found on the submucosa. We separated the mass along its edge and stanched the bleeding with electrocoagulation. The wound was closed with titanium clips after the tumor was removed. Then, the patient was treated with fasting, anti-infection, acid suppression, and fluid replacement for 1 week. The patient fully recovered, and was discharged.

The patient was followed up for 3 months after operation and recovered well. His symptoms of GI tract have been relieved.

**Figure 3:**
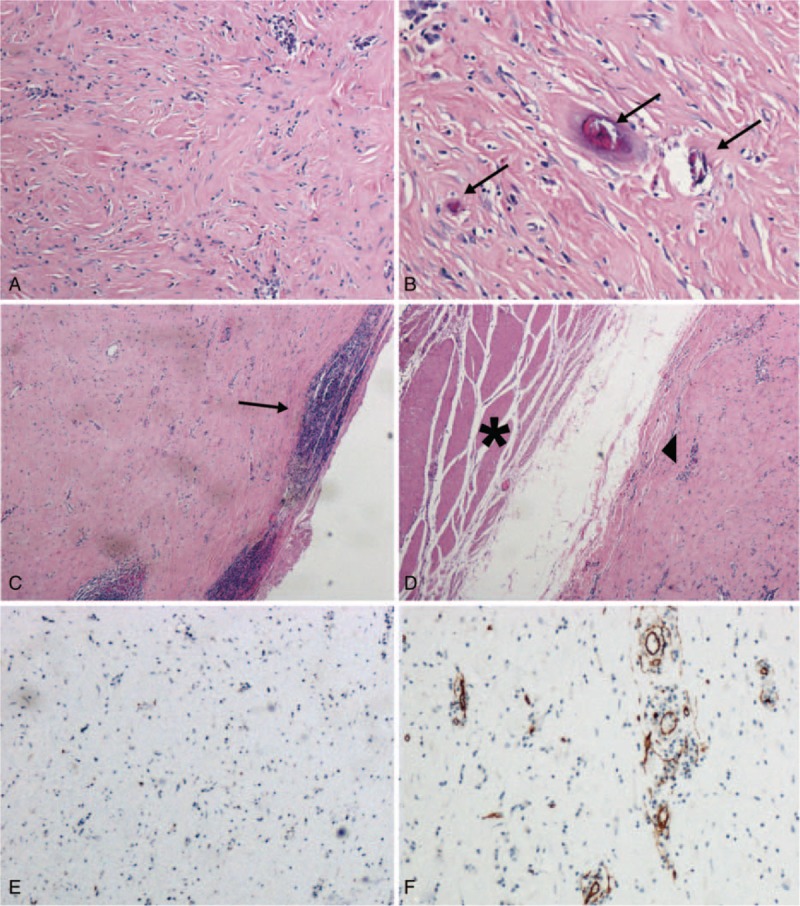
Pathologic findings of the tumor. The tumor was mainly composed of abundant hyalinized collagen infiltrated with chronic inflammatory cells (A). Scattered calcifications were observed in the lesion (B). Lymphocytes infiltrated and formed germinal centers, and prominent lymphoid cuff was found (C). The tumor border (as shown by the asterisk) was distinct without infiltrating the muscular layer (as shown in the triangle) of the gastric wall (D). The immunohistochemical staining showed that the tumor cells were negative for CD117 (E). The CD34 staining was positive for blood vessels but negative for tumor cells (F).

The tumor was well-circumscribed, unencapsulated mass with a homogenous grey-white cut surface. Microscopically, the tumor was mainly composed of abundant hyalinized collagen infiltrated with chronic inflammatory cells (Fig. 3A). Scattered calcifications were observed in the lesion (Fig. 3B). Lymphocytes infiltrated and formed germinal centers, and prominent lymphoid cuff was found (Fig. 3C). The tumor border was distinct without infiltration of the muscular layer of the gastric wall (Fig. 3D). The immunohistochemical staining showed that the tumor cells were negative for CD117, a distinctive marker of GIST (Fig. 3E). The CD34 staining was positive for blood vessels but negative for tumor cells (Fig. 3F). According to the morphological and immunohistochemical features, the diagnosis of gastric CFT was made. This study was approved by the Ethical Committee of Second Military Medical University Gongli Hospital. Informed consent was given by the patient.

## 3 Discussion

Patients with CFTs could present with abdominal discomfort, ulceration, or obstructive symptoms, and are incidentally identified. CFT was thought to be related to inflammatory myofibroblastic tumor and IgG4-related sclerosing disease, but this hypothesis remains unsubstantiated.^[7]^ CT, endoscopy, or MRI can reveal the tumor size and localization, but the final diagnosis is made after histopathological examination.

GI tract CFTs differ from soft CFTs in features such as perivascular accentuation of lymphoid cells and prominent lymphoid cuff, which are not observed in soft CFTs. Gastric CFTs should be differentiated from other spindle cell mesenchymal lesions such as GIST, schwannomas, inflammatory fibroid polyps, and hyalinized leiomyomas. The cells of GIST lack typical features of CFT such as hyalinized stroma, lymphocytic infiltration, and lymphocytic cuff, but are positive for CD117, CD34, and DOG-1.^[8]^ Like CFT, gastric schwanommas also have prominent lymphoid cuff, but higher cellularity. The tumor cells of schwannoma are typically positive for S100 protein and Glial Fibrillary Acidic Protein, but negative for CD34 and Smooth Muscle Actin by immunohistochemistry.^[9]^ In contrast to GI tract CFTs, inflammatory fibroid polyps have higher cellularity and frequently display whorled proliferations of spindle cells around vessels and variable numbers of intralesional eosinophils.^[10]^ Leiomyomas of the GI tract were composed of perpendicularly intersecting fascicles of brightly eosinophilic spindle cells with bland blunted nuclei, which expressed markers associated with smooth muscle differentiation such as actin, desmin, h-caldesmon, and calponin.^[11]^

CFTs are benign mesenchymal tumors, and local excision alone can cure the disease in most cases. There were few reported recurrent cases, most of which occurred in the neck of infants.^[3]^ The World Health Organization classification of soft and bone tumor pathology, and genetics classified it into a new type of benign tumor derived from fibroblasts/myofibroblasts and named it CFT.^[12]^

In conclusion, as CFT is a rare mesenchymal tumor that typically occurs in the stomach, it can often be misdiagnosed as other GI tract tumors, especially GIST. Given its benign biological behavior, ESD surgery is suitable and effective for the treatment of CFT.
